# CircRNF220, not its linear cognate gene RNF220, regulates cell growth and is associated with relapse in pediatric acute myeloid leukemia

**DOI:** 10.1186/s12943-021-01395-7

**Published:** 2021-10-26

**Authors:** Xiaodan Liu, Xiaoping Liu, Mansi Cai, Ailing Luo, Yingyi He, Sha Liu, Xiaohong Zhang, Xu Yang, Ling Xu, Hua Jiang

**Affiliations:** 1 Division of Birth Cohort Study, Guangzhou Women and Children’s Medical Center, Guangzhou Medical University, Guangzhou, China; 2 Department of Hematology/Oncology, Guangzhou Women and Children’s Medical Center, Guangzhou Medical University, 9 Jinsui Road, Zhujiang Newtown, Tianhe District, Guangzhou, 510623 Guangdong China; 3 Institute of Pediatrics, Guangzhou Women and Children’s Medical Center, Guangzhou Medical University, Guangzhou, China

**Keywords:** Circular RNA, RNF220, miR-30a, Acute myeloid leukemia

## Abstract

**Background:**

Circular RNAs (circRNAs) constitute a family of transcripts with unique structures and have been confirmed to be critical in tumorigenesis and to be potential biomarkers or therapeutic targets. However, only a few circRNAs have been functionally characterized in pediatric acute myeloid leukemia (AML).

**Methods:**

Here, we investigated the expression pattern of circRNAs in pediatric AML using a circRNA microarray. The characteristics, potential diagnostic value, and prognostic significance of circRNF220 were evaluated. A series of functional experiments were performed to investigate the role of circRNF220 in primary pediatric AML cells. Then we investigated the aberrant transcriptional networks regulated by circRNF220 in primary AML cells by RNA-seq. Furthermore, biotin RNA pulldown assays were implemented to verify the relationship between circRNF220 and miR-30a.

**Results:**

We identified a circRNA, circRNF220, which was specifically abundant in and accumulated in the peripheral blood and bone marrow of pediatric patients with AML. It could distinguish AML from ALL and other hematological malignancies with high sensitivity and specificity. Significantly, circRNF220 expression independently predicted prognosis, while high expression of circRNF220 was an unfavorable prognostic marker for relapse. Furthermore, we characterized the function of circRNF220 and found that circRNF220 knockdown specifically inhibited proliferation and promoted apoptosis in AML cell lines and primary cells. Mechanistically, circRNF220 may act as an endogenous sponge of miR-30a to sequester miR-30a and inhibit its activity, which increases the expression of its targets MYSM1 and IER2 and implicated in AML relapse.

**Conclusions:**

Collectively, these findings demonstrated that circRNF220 could be highly efficient and specific for the accurate diagnosis of pediatric AML, with implications for relapse prediction.

**Supplementary Information:**

The online version contains supplementary material available at 10.1186/s12943-021-01395-7.

## Introduction

Acute myeloid leukemia (AML) is a relatively rare malignancy among childhood malignancies, accounting for less than one-fifth of acute leukemia cases diagnosed in childhood. However, it remains a challenging disease with a poor outcome compared with that of pediatric acute lymphoblastic leukemia (ALL) [1]. Despite enormous progress in AML diagnosis and treatment during the past decades, approximately 40% of children with AML experience short-term relapse [2, 3]. In AML, some risk factors, such as age, cytogenetic characteristics, the white cell count (WBC), and minimal residual disease (MRD), are currently used for prognosis [4–7]. However, these assessed covariates are of limited predictive value and have been commonly reported in adults. Few have been assessed in pediatric populations of patients [8, 9]. Therefore, not only accurate and timely diagnosis but also valuable prognostic biomarkers and novel therapeutic algorithms are critical for the clinical management of pediatric AML [10, 11].

Circular RNAs (circRNAs) are covalently closed nonlinear RNA molecules that are produced from pre-mRNA backsplicing and have been considered faulty splicing products for several decades [12]. However, it has become clear that circRNAs are functional entities [13]. Notably, the majority of circRNAs consist of varying numbers of constitutive exons and are extremely stable RNA molecules with cell type- or tissue type-specific expression patterns [14]. CircRNAs play a potential role as biomarkers in a variety of cancers owing to their biological characteristics as well as their involvement in tumorigenesis through diverse mechanisms, such as microRNA (miRNA) sponging, RNP binding, or acting as templates for translation [15–18].

Emerging evidence has shown that the roles of a few circRNAs are well established in AML [19]. For instance, circ-ANXA2 is upregulated in AML, and its knockdown (KD) suppresses the proliferation, enhances the apoptosis and increases the chemosensitivity of THP-1 and KG-1 cells by acting as an effective microRNA sponge of miR-23a-5p and miR-503-3p [20]. CircPAN3 was validated to mediate doxorubicin resistance in AML cells by targeting the miR-153-5p/miR-183-5p/XIAP axis and enhancing autophagy activity [21]. More recently, Sun et al. reported that circMYBL2 promotes the proliferation of FLT3-ITD–positive AML cells by directly interacting with the PTBP1 protein *in* *vitro* and *in* *vivo* [22]. It is worth noting, however, that much less is known about the regulatory influence of circRNAs in pediatric AML.

Here, we sought to investigate the regulatory roles played by circRNAs in pediatric AML. Our screening strategy identified that circRNF220, a circRNA derived from the RING domain E3 ubiquitin ligase gene RNF220, was specifically upregulated in pediatric AML and was required for AML progression. CircRNF220 KD significantly impeded the activities of primary pediatric AML cells. Furthermore, we revealed that circRNF220 performed its regulatory functions as a competing endogenous RNA by binding miR-30a and then regulating the expression of downstream genes Myb like, SWIRM and MPN domains 1(MYSM1) and immediate early response 2(IER2). Importantly, we showed that circRNF220 not only outperformed current markers in terms of its high sensitivity, specificity and diagnostic simplicity but also demonstrated prognostic value for early recurrence by evaluation in bone marrow (BM) and even peripheral blood (PB) samples.

## Methods

### Patients and samples

Diagnostic BM or PB samples were collected from 149 pediatric patients (1 to 14 years of age) with de novo AML of French-American-British (FAB) classification M1-M7 enrolled in this study between January 2015 and May 2020 at the Guangzhou Women and Children’s Medical Center of Guangzhou Medical University who had available pretreatment and post chemotherapy cell samples. The demographic characteristics of the AML patients are summarized in supplementary Table S1. All patients received intensive, response-adapted double induction and consolidation therapy, and the details of the treatment regimen have been reported previously [23]. In addition, BM samples from 86 patients with ALL, 28 patients with idiopathic thrombocytopenic purpura (ITP), and 33 patients with other hematological diseases, including myelodysplastic syndrome (MDS), chronic myeloid leukemia (CML), juvenile myelomonocytic leukemia (JMML), anemia, lymphoma, and thrombocytopenia, were also analyzed. Detailed information on these patients is available in supplementary Table S2. Informed patient consent for the study and accompanying scientific investigations was obtained from the participants’ parents or guardians. The research was conducted after approval by the Ethics Committee of Guangzhou Women and Children’s Medical Center according to the principles of the Declaration of Helsinki.

### CircRNA microarray

A circRNA microarray (Arraystar Human circRNAs chip, ArrayStar) containing more than 5000 probes specific for splice sites in human circRNAs was used in this study. After hybridization, 5 pediatric AML samples (pooled) and 5 healthy donor BM samples (pooled) were examined using the circRNA microarray provided by Kangcheng Bio-Tech Inc. R software was used to process the subsequent data after quantile normalization. Differentially expressed circRNAs were identified through volcano plot filtering and fold change filtering. CircRNAs with a fold change ≥ 2.0 or ≤ 0.5 and a *P*-value < 0.05 were identified as significantly differentially expressed circRNAs. We further confirmed the microarray results for 10 randomly selected circRNAs by qRT-PCR in samples from 10 newly diagnosed AML patients and healthy controls.

### RNA isolation, reverse transcription, and real-time quantitative polymerase chain reaction (qRT-PCR)

Total RNA from whole-cell lysates or the nuclear and cytoplasmic fractions was extracted from BM and PB samples using TRIzol® reagent (Life Technologies). To quantify the amounts of mRNAs and circRNAs, cDNA was synthesized from 500 ng of RNA with PrimeScript RT Master Mix (Takara, Dalian, China). QPCR analyses were performed using SYBR Premix Ex Taq II (Takara) and a LightCycler® 480 Real-Time PCR System (Roche, Basel, Switzerland). Specifically, divergent primers annealing at each side of the backsplice junction were used to determine the abundances of circRNAs. The expression levels were calculated using the 2^−△△*C*t^ method, and GAPDH was used as the internal standard. All assays were performed in triplicate. The sequences of all primers used are available (Supplementary Table S3).

### RNA fluorescence in situ hybridization (RNA-FISH)

RNA FISH was performed on AML cells and 293 T cells using an RNA Fluorescence In Situ Hybridization Kit (Exonbio Lab, Guangzhou, China) in accordance with the manufacturer’s protocol. Probe sequences designed by Exonbio Lab are listed in Supplementary Table S3. AML cells or 293 T cells at 85–95% confluence were fixed with 4% paraformaldehyde for 12 min. After prehybridization, cells were incubated with probes in hybridization buffer at 37 °C overnight. After hybridization, slides were washed in 2 × SSC with 0.5% Tween 20 two tough times for 15 min each at room temperature. Nuclei were counterstained with DAPI (Invitrogen, Carlsbad, CA, USA) for 10 min. Images were acquired on a Leica TCS SP8 confocal microscope (Leica Microsystems, Mannheim, Germany).

### Cell culture and treatments

Human leukemia cell lines (HL-60, THP-1, and K562) and the human embryonic kidney cell line HEK-293 T were purchased from the American Type Culture Collection and maintained in RPMI 1640 medium or DMEM (Invitrogen) supplemented with 10% fetal bovine serum (FBS). Cells were cultured at 37 °C in 5.0% CO_2,_ and all cell lines were routinely tested for mycoplasma contamination. AML BM mononuclear cells were prepared by Ficoll density centrifugation at 600 × g for 30 min, and then were thawed and plated in MEMα (Gibco) supplemented with 10% FBS, 20 ng/ml hTPO, 20 ng/ml hIL-3, 20 ng/ml hG-CSF, and 1 × penicillin/streptomycin.

AML cells were exposed to 2 μg/mL actinomycin D (Sigma-Aldrich, Saint Louis, MO, USA) to block transcription for 4, 8, 12, 24, and 48 h. Then, the cells were harvested, and the stability of the circRNF220 and RNF220 mRNAs was analyzed using qRT-PCR.

All in vitro studies were repeated three times in AML cell lines, or performed in more than three primary AML cases BM cells.

### RNase R assay

Total RNA was treated with RNase R. Briefly, 5 μg of total RNA was exposed to 2 U/μg RNase R (Epicenter Biotechnologies) at 37 °C for 30 min, and RNase-free water was used as a control (Mock). Digested RNA was subsequently purified using an RNeasy MinElute Cleanup Kit (Qiagen). The RNA concentrations of the treated samples were determined, and 1 μg of treated RNA was used for qRT-PCR.

### Nuclear and cytoplasmic fractionation

The nuclear and cytoplasmic fractions were extracted according to the manufacturer’s protocol using NE-PER Nuclear and Cytoplasmic Extraction Reagents (Thermo Scientific). The effectiveness of nuclear and cytoplasmic separation was assessed by determining the protein levels of histone H3 and GAPDH, which are specifically expressed in the nucleus and cytoplasm, respectively.

### Small interfering RNAs (siRNAs), vector construction, and cell electroporation

Small interfering RNAs (siRNAs) against circRNF220 (siRNA-circRNF220) and the negative control RNA duplex (siRNA-NC) were purchased from RiboBio, Guangzhou, China. The miR-30a mimics (miR-30a) or its inhibitor (inhibitor-miR-30a) and scrambled oligonucleotides (miR-NC or inhibitor-NC) were purchased from GenePharma Biotech (Shanghai, China).

The plasmid for circRNA overexpression (OE) was constructed using the pcDNA3.1( +) circRNA Mini Vector, which was produced by the Forevergen Company (Guangzhou, China). The region spanning the second exon of the RNF220 gene that constitutes the circRNA was PCR amplified from cDNA and seamlessly cloned between the splicing signals AG and GT, which are surrounded by the minimal introns that facilitate the generation of circRNF220 (pcDNA3.1-circRNF220). The construct was verified by sequencing.

In addition, based on the expression levels of circRNF220 in AML cells, we selected the AML cell lines with comparatively low circRNF220 expression (HL-60, THP-1 and K562) to perform gain-of-function experiments. Primary BM cells expressing comparatively high levels of circRNF220 were used to perform loss-of-function experiments. Unless otherwise noted, the plasmids and siRNAs were delivered into cultured AML cells by electroporation as we reported before [24]. The exception was primary AML cells (3 × 10^5^ cells/sample), which were transfected with 100 pmol of the siRNAs in T buffer using the Neon® Transfection System (Invitrogen) with settings of 1400 V, 10 ms and 1 pulse.

The transfected cells were collected at different time points after electroporation for biological functional analyses and for RNA extraction.

### Lentiviral constructs and lentiviral transduction of patient AML samples

The circRNF220 KD lentivirus (LV-sh-circRNF220) was produced by GeneChem Company (Shanghai, China). The shRNAs only specifically targeted the head-to-head splicing junction of circRNF220. The particular sequences were synthesized and cloned into the *Age*I and *EcoR*I sites in the 5’LTR of MCS-CBh-gcGFP, and the integrity of the insert was confirmed by sequencing.

AML BM cells were transduced with retroviral vectors according to the protocol. Briefly, LV-sh-circRNF220 lentiviral particles were centrifuged for 2 h at 1,200 × g in 24-well plates precoated with 100 μg/ml RetroNectin (Takara, Dalian, China). Three days later, the cells were sorted to separate GFP + populations for subsequent experimental studies.

### CCK-8 assay

Cell proliferation was assessed using a Cell Counting Kit-8 (Dojindo Molecular Technologies, China), and samples were evaluated in a microplate reader (Varioskan Flash, Thermo Scientific) as described previously [24].

### Cell cycle analysis

Treated AML cells were stained with PI (KeyGEN, Nanjing, China) containing RNase A for 30 min at 37 °C. Cells were analyzed for DNA content by fluorescence activated cell sorting (FACS) (BD Bioscience, San Jose, CA, USA) with ModFit software used for data analysis, as described previously [24].

### AML cell differentiation

After treatment with 25 ng/mL phorbol-12-myristate 13-acetate (PMA, Sigma-Aldrich) for 2 days, THP-1 cells were reseeded in fresh medium without PMA for 2 more days to allow cell recovery. Then, monocytic differentiation was evaluated based on the cell morphology on Wright-Giemsa-stained slides and by FACS analysis. For FACS analysis, cells were stained with anti-human CD14-PE (eBioscience, San Diego, CA, USA) and anti-human CD11b-APC (Invitrogen) monoclonal antibodies. The data were analyzed using FlowJo software (BD Biosciences).

### Apoptosis assay

Treated AML cells were stained with Annexin V/PI (KeyGEN) and subsequently analyzed by FACS. We defined all Annexin V^+^ cells as apoptotic cells, including the early apoptotic cells and the late apoptotic cells. Additionally, we performed terminal deoxynucleotidyl transferase (TdT)-mediated dUTP nick-end labeling (TUNEL) to detect apoptotic K562 cells using the corresponding kit (Beyotime).

### RNA sequencing (RNA-seq) analysis

RNA-seq analysis was performed by Capital Bio Technology (Beijing, China). Before preparation of the RNA-seq libraries, total RNA (1 μg) was treated with a RiboMinus Eukaryote Kit (Qiagen, Valencia, CA) to remove rRNA. Strand-specific RNA-seq libraries were prepared using a NEBNext Ultra RNA Library Prep Kit for Illumina (NEB, Beverly, MA) following the manufacturer’s instructions. The libraries were quality controlled with a 2100 Bioanalyzer chip (Agilent, Santa Clara, CA) and sequenced on the HiSeq 2000 platform (Illumina, San Diego, CA) with 100 bp paired-end reads.

RNA sequencing reads were mapped to the GRCh38.p7 assembly using STAR aligner version 2.5.2b with Ensembl annotations. Data analysis was performed with R software with a low-stringency filtering scheme of a 1.5-fold change in the expression level after MAS5 normalization of all datasets. To investigate the global effect of circRNF220 on AML cells, we used KOBAS 3.0 (http://kobas.cbi.pku.edu.cn/index.php) for pathway analysis of the gene expression profile data for AML cells transfected with siRNA-circRNF220. Finally, Gene Set Enrichment Analysis (GSEA) v.2.0 was used to analyze a preranked list based on the DESeq2 *t*-statistic using preassembled gene sets from MSigDB v5.2.

### Circular RNA–microRNA analysis

To predict the miRNA and AGO protein binding sites in circRNF220, we used the bioinformatics databases CircNet [25] and Circular RNA Interactome [26]. By combining these data with GSEA data, five miRNAs whose expression was significantly correlated with that of circRNF220 and were predicted to bind within the AGO sites were selected for downstream investigation.

### RNA pulldown assay

A pulldown assay was performed as previously reported [27]. The biotin-labeled circRNF220 probe was synthesized by Exonbio Lab (Guangzhou, China). A total of 1 × 10^7^ circRNF220-overexpressing AML cells were harvested and lysed. The circRNF220 or oligo probe was incubated with streptavidin-coupled dynabeads at room temperature for more than 30 min to generate probe-bound dynabeads. After the treated beads were washed, the RNA complexes bound to the beads were eluted and disrupted with lysis buffer and proteinase K before analysis by qRT-qPCR. The biotinylated probe sequences used in this study are listed in Supplementary Table S3.

### Statistical analysis

Differences in mRNA, circRNA, or miRNA expression between two groups were analyzed using the Mann–Whitney U test for independent unpaired samples and the Wilcoxon test for paired samples. For comparisons among more than two groups, the Kruskal–Wallis test was performed first, and the Bonferroni correction for multiple comparisons was then applied. The χ^2^ or Fisher’s exact test was used for analysis of categorical variables, and the Spearman correlation coefficient (*r*) was determined to analyze correlations. Receiver operating characteristic (ROC) analysis was applied to evaluate the power of circRNF220 expression as a prognostic marker in AML, and the value of (Ct_circRNF220_-Ct_GAPDH_) served as the estimated optimal diagnostic threshold. The area under the curve (AUC), sensitivity, and specificity were also calculated. The Kaplan–Meier method was used to analyze relapse-free survival (RFS), and the log-rank test was then performed to compare the outcomes among subgroups. Finally, a Cox proportional hazard regression model was used for univariate and multivariate analysis of prognostic factors. All calculations were performed using GraphPad Prism software and R software with a two-sided *P* < 0.05 considered to indicate a statistically significant difference.

## Results

### Profiling of circRNAs in pediatric primary AML

**Fig. 1:**
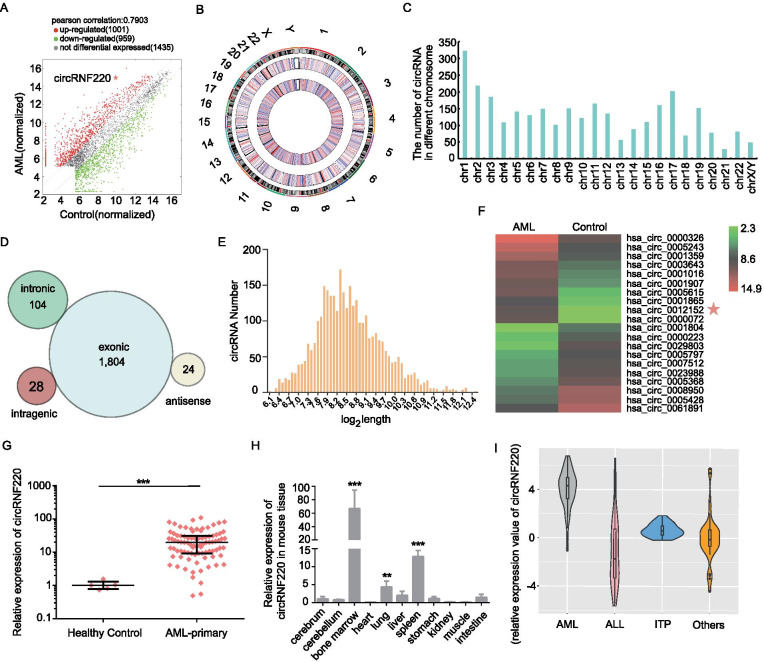
Profiling of circRNAs in bone marrow from primary pediatric AML patients and normal controls. **A** A scatter plot showing the variation in pediatric AML patients and controls. The red plot indicates circRNAs with greater than 2.0-fold upregulation between the two compared samples, while the green plot indicates the downregulated circRNAs. The star symbol denotes circRNF220. **B**-**C** The distribution of differentially expressed circRNAs on chromosomes. **D** Genomic origins of human circRNAs from primary pediatric AML patients. **E** The length distribution of circRNAs in pediatric AML. **F** A heat map showing the top 10 upregulated and downregulated circRNAs in the case group *vs.* the control group. Each row corresponds to a circRNA. High expression levels are indicated by red, and lower levels are indicated by green. **G** The expression levels of circRNF220 in AML patients were significantly higher than those in the corresponding controls. **H** qRT–PCR analysis of circRNF220 expression in various mouse organs and tissues. **I** Violin plot showing the relative abundances of circRNF220 in several hematologic malignancies. Data are expressed as log2 fold change values. The centerline in the box plot indicates the median, and the black dot indicates an abnormal value. AML, acute myeloid leukemia; ALL, acute lymphocytic leukemia; ITP, idiopathic thrombocytopenic purpura; Others, diseases including anemia, thrombocytopenia, chronic myeloid leukemia, lymphoma, juvenile myelomonocytic leukemia, and myelodysplastic syndrome. **P < 0.01, *** *P* < 0.001

To investigate the circRNA expression profile in pediatric primary AML, a high-throughput human circRNA microarray analysis was conducted using BM samples from 5 pediatric AML patients (pooled) and 5 normal individuals (control). The scatter plot visualization showed that circRNA expression levels were variable and distinguishable among the groups (Fig. 1A). A total of 1960 circRNAs were differentially expressed between the AML and control samples, with 1001 circRNAs found to be upregulated (fold change ≥ 2.0) and 959 downregulated (fold change ≤ 0.5). Chromosomes 1 and 2 hosted the most significant number of differentially expressed circRNAs (Fig. 1B-C), and most differentially expressed circRNAs in AML were transcribed from exons (Fig. 1D). The length of most dysregulated circRNAs was less than 1500 nucleotides, and the median length was 372 nucleotides (Fig. 1E). Then, the top 10 upregulated and downregulated circRNAs were identified by hierarchical clustering (Fig. 1F). In summary, we observed striking differences in circRNA expression in pediatric primary AML, supporting the importance of studying the role of circRNAs in myeloid leukemogenesis.

We confirmed the microarray results by qRT-PCR using the same cohort of patients and control samples used in the microarrays (Supplementary Fig S1A). CircRNF220 (hsa_circ_0012152 is located in the RNF220 gene; thus, we named this circRNA circRNF220) was the best candidate identified in the extensive sample validation process (Fig. 1G). We found that circRNF220 expression was consistently and significantly increased in primary AML patients compared with healthy controls. Of note, circRNF220 is commonly expressed in various tissues and extremely enriched in hematopoietic tissues, such as the BM, spleen and lung, in mice (Fig. 1H). Moreover, among the childhood leukemias and other hematological system diseases (including ALL, MDS, JMML, CML, ITP, anemia, lymphoma, and thrombocytopenia) that we investigated, circRNF220 was highly expressed in pediatric AML (Fig. 1I).

### Identification and validation of circRNF220 in AML

**Fig. 2:**
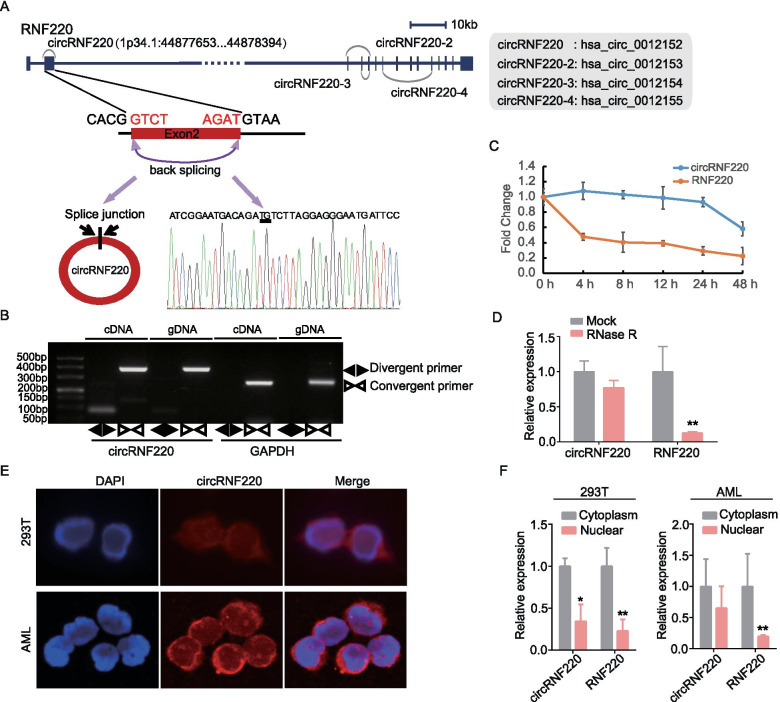
Identification and characterization of circRNF220 in pediatric AML. **A** Structures of the RNF220 genomic locus and transcript. CircRNF220 is produced by exon 2. The junction point of circRNF220 was identified by Sanger sequencing. **B** Divergent primers were used to amplify circRNF220 from cDNA but not genomic DNA (gDNA). Convergent primers amplified both circRNF220 and the linear GAPDH RNA in cDNA and gDNA. **C** qRT–PCR analysis of the abundances of circRNF220 and RNF220 mRNA in AML cells treated with actinomycin D at the indicated time points. **D** qRT–PCR analysis of the abundances of circRNF220 and RNF220 mRNA in AML cells treated with RNase R, with normalization to the value measured in the mock treatment sample. **E** RNA fluorescence in situ hybridization analysis of circRNF220 in 293 T and AML cells. Nuclei were stained with 4,6-diamidino-2-phenylindole (DAPI). **F** qRT–PCR data indicating the abundances of circRNF220 and RNF220 mRNA in the cytoplasm and nucleus of 293 T and AML cells. The abundances of circRNF220 and RNF220 mRNA were normalized to the values measured in the cytoplasm. The data in (**C**-**D**) and (**F**) are the mean ± SD, which carried out in cell lines for three times, or in more than 3 primary pediatric AML cases BM cells. *P < 0.05, **P < 0.01

Next, we verified the existence of circRNF220 in the circBase database and circBank database. The genomic structure showed that circRNF220 is located at 1p34.1 and contains a relatively large second exon (742 bp) derived from the RNF220 gene flanked by long introns on the right side (Fig. 2A). The distinct product of the expected size was amplified using outward-facing primers and confirmed by Sanger sequencing (Fig. 2A). In addition to circRNF220, three other circRNA isoforms were identified in the RNF220 gene locus (we termed these circRNF220-2, circRNF220-3, and circRNF220-4; Fig. 2A). CircRNF220 is the predominant circRNA isoform, as evidenced by our verification (Supplementary Fig S1B). To further characterize circRNF220, divergent primers were designed to amplify the circular transcripts, and convergent primers were used to detect the linear transcripts in both cDNA and gDNA. PCR results indicated that the circular form was amplified from cDNA but not gDNA using the divergent primers but from both cDNA and gDNA using the convergent primers (Fig. 2B).

We then investigated the stability and localization of circRNF220 in primary AML cells. Total RNA was harvested at the indicated time points after treatment with actinomycin D, an inhibitor of transcription. Analysis of circRNF220 and RNF220 mRNA revealed that the circRNA isoform was highly stable, with a transcript half-life exceeding 48 h, compared to the linear transcript, which exhibited a half-life of 4 h (Fig. 2C). Resistance to digestion with the exonuclease RNase R further confirmed that circRNF220 is circular in form (Fig. 2D). In addition, qRT–PCR analysis of nuclear and cytoplasmic circRNF220 expression and the results of FISH for circRNF220 demonstrated that circRNF220 preferentially localized in the cytoplasm and that, of course, a moderate proportion was localized in the nucleus (Fig. 2 E–F). Taken together, our results showed that circRNF220 is an abundant and stable circRNA expressed in pediatric AML cells.

### The potential diagnostic and prognosis predicting value of circRNF220 in pediatric AML

**Fig. 3:**
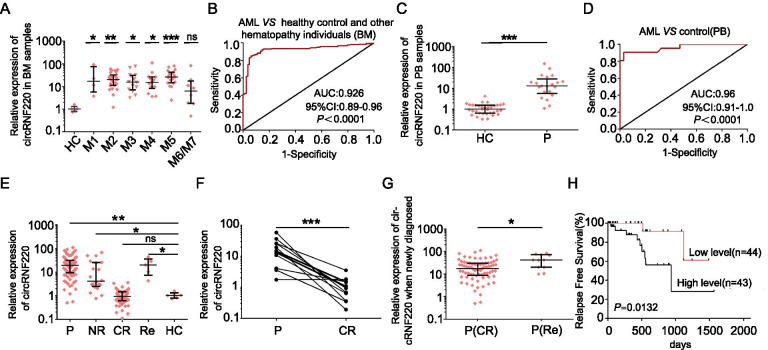
CircRNF220 had potential diagnostic value and was related to the prognosis of pediatric AML patients. **A** The expression level of circRNF220 in patients with different subtypes of pediatric AML from M1 to M7. Every subtype was compared to the healthy control group. **B** Receiver operating characteristic (ROC) analysis for circRNF220 in the BM of AML patients. The area under the ROC curve (AUC) for distinguishing AML patients from healthy controls and patients with other hematological diseases was 0.9260. **C** qRT-PCR validation of circRNF220 expression in PB samples from patients with pediatric AML. **D** ROC analysis of circRNF220 in AML patient PB samples. The AUC for distinguishing patients with AML from normal controls was 0.9601. **E** CircRNF220 expression was measured by qRT-PCR in samples from 187 AML patients with different stages of disease (P, n = 87; NR, n = 20; CR, n = 75; Re, n = 5). **F** The average expression levels of circRNF220 before and after therapy (n = 13) in the paired samples from pediatric AML patients. **G** A total of 87 primary AML patient samples were divided according to the clinical outcomes of the corresponding patients. Patients who relapsed had significantly higher circRNF220 expression levels than those who achieved CR after therapy. **H** Relapse-free survival (RFS) curves for a cohort of 87 AML patients. The patients were dichotomized based on the median expression level of circRNF220 at diagnose. Statistical differences between the curves were calculated by using the log-rank test, and the two-sided *P*-value is indicated below the graph. HC, healthy control; BM, bone marrow; PB, peripheral blood; P, primary; NR, nonremission; CR, complete remission; Re, relapse; P (CR), primary patient with complete remission; P (Re), primary patient with relapse. * *P* < 0.05, **P < 0.01, *** *P* < 0.001. ns: not significant

Given the tremendous diagnostic and therapeutic role of circRNAs in leukemia, we explored the clinical value of circRNF220. We first found that circRNF220 expression was consistently and significantly increased in patients with almost all AML subtypes (except M6/M7) compared with healthy controls (Fig. 3A), while its host gene RNF220 was not (Supplementary Fig S1C), although there was a weak correlation between the expression levels of circRNF220 and RNF220 (Supplementary Fig S1D). However, the transcript abundance of circRNF220 was more than 100 times that of its linear counterpart, RNF220 (Supplementary Fig S1E). Based on the median level of circRNF220 expression in the cohort, we divided the pediatric AML patients into high and low expression groups to determine whether circRNF220 expression correlates with clinic pathological characteristics. However, there were no significant differences in the available pathological data (Table 1), which implies that circRNF220 might function in an independent manner.

**Table 1:** Comparison of clinical manifestation and laboratory features between pediatric primary AML patients with low and high circRNF220 expression

Characteristics	circRNF220 expression		*P* value^#^
	Low(n=44)	High(n=43)	
Median age(range), year	6(1-14)	6(1-13)	0.957
Gender(male/female)	27/17	25/18	0.759
Median WBC(range), (×10^9^/L)	11.95(1-608.8)	20(0.6-671)	0.371
Median hemoglobin(range), g/L	81.5(45-130)	79(39-136)	0.665
Median platelets(range), (×10^9^/L)	56.5(10-713)	53(9-431)	0.530
Bone marrow blasts( range), %	53(2-91)	59(2-93)	0.766
Peripheral blood blasts( range), %	27(0-87)	31(0-92%)	0.497
FAB classification			0.085
M1	2(4.5%)	2(4.7%)	
M2	11(25%)	17(39.5%)	
M3	7(15.9%)	5(11.6%)	
M4	9(20.5%)	6(14%)	
M5	7(15.9%)	12(28%)	
M6	0(0%)	1(2.3%)	
M7	6(13.6%)	0(0%)	
No data	2(4.5%)	0(0%)	
Clinical Risk^†^			0.752
Favorable	7(15.9%)	8(18.7%)	
Intermediate	14(32.6%)	16(37.2%)	
Poor	23(52.3%)	19(44.2%)	
Cytogenetic aberrations			0.508
normal	11(25%)	11(25.6%)	
t(8;21)	1(2.3%)	3(7%)	
t(15;17)	4(9.1%)	1(2.3%)	
11q23	4(9.1%)	4(9.3%)	
others	1(2.3%)	4(9.3%)	
complex	8(18.2%)	5(11.6%)	
No data	15(34.1%)	15(34.9%)	
Gene mutation			
CEBPA(+/-)	3/41	1/42	0.625
FLT3-IDT(+/-)	6/38	2/41	0.280
c-KIT(+/-)	4/40	2/41	0.694
NRAS or KRAS(+/-)	2/42	2/41	0.625
Others(+/-)	6/38	3/40	0.504

*Abbreviations*: *AML *Acute myeloid leukemia, *WBC *White blood cells, *FAB *French-American-British, *CR *Complete-remission. +, mutation; -, wild type

^#^*P* value from two-sided χ2 tests for categorical variables and from two-sided Student’s t tests for continuous variables

^†^The cytogenetic risk group is defined according to Medical Research Council criteria

Next, we performed ROC curve analysis to examine the potential diagnostic value of circRNF220 expression in distinguishing individuals with AML from healthy controls and individuals with other hematological diseases. The estimated optimal diagnostic threshold was 9.525, which yielded a sensitivity of 85.25% (95% CI, 80.16–89.45) and a specificity of 93.98% (95% CI, 86.5–98.02). The AUC was 0.926 (95% CI, 0.8948–0.9572) (Fig. 3B). In this subgroup analysis, the AUCs were 0.9735, 0.9209 and 0.9274 for distinguishing individuals with AML from healthy controls, individuals with ALL and individuals with other hematological diseases, respectively (Supplementary Fig S2A-C).

Intriguingly, the results indicated that circRNF220 also had significantly higher expression in PB samples from patients with AML (Fig. 3C) and that circRNF220 in PB samples showed a strong positive correlation with circRNF220 expression in BM samples (Supplementary Fig S2D). Furthermore, ROC curve analysis was performed to examine the potential diagnostic value of circRNF220 expression in PB samples. The results showed that the AUC was 0.9601 (Fig. 3D), and the cutoff value was 9.295 with a sensitivity of 90.48% and specificity of 97.06% for distinguishing individuals with AML from healthy individuals, indicating that circRNF220 in either BM or PB samples has considerable potential as a diagnostic biomarker.

To identify the biological mechanisms affecting AML prognosis, we validated circRNF220 expression in AML patients at different treatment stages. A much higher expression level of circRNF220 was found in newly diagnosed AML patients than in healthy controls (Fig. 3E). Moreover, circRNF220 expression in patients who did not achieve remission was slightly lower than that in the primary AML group. CircRNF220 expression decreased dramatically in patients who achieved complete remission (CR) after treatment, showing no difference compared to that in the control group (Fig. 3E). The Wilcoxon signed-rank test results showed that circRNF220 expression was sharply decreased in almost all paired of the CR samples (Fig. 3F). However, in relapsed-refractory patients, upregulated circRNF220 expression was again observed (Fig. 3E). These results demonstrated the dynamic expression of circRNF220 according to the progressive stage of AML, suggesting that it might have a complicated role in leukemogenesis and relapse.

After a median follow-up of 475 days (range from 35 to 1566 days), among the 87 primary AML patients initially enrolled, 11 patients (12.6%) relapsed, and the RFS rate was 87.4%. After follow-up with these patients, we found that circRNF220 expression in the patients who relapsed (n = 11) was nearly 2.5-fold higher at the time of diagnosis than that in the remaining 76 patients with continuous CR (Fig. 3G). The results further indicated that circRNF220 expression was related to leukemia relapse; thus, we hypothesized that circRNF220 could be a biomarker for predicting relapse in pediatric patients with AML.

When the full set of 87 samples was divided into different expression groups, we observed that patients with high circRNF220 expression at the time of diagnosis had a lower RFS rate (79.07%) than patients with low circRNF220 expression, who had an RFS rate of 95.45% (Fig. 3H). Next, we used univariate Cox regression analysis to evaluate the impact of frequently used clinical prognostic factors. Patients with higher circRNF220 expression had a greater risk of recurrence (*P* = 0.027, HR = 5.749; 95% CI, 1.219 to 27.101) than patients with lower circRNF220 expression in univariate analysis. Furthermore, patients with higher circRNF220 expression had a 5.866-fold higher risk of relapse (*P* = 0.026, 95% CI 1.23 to 27.969) in multivariate Cox regression analysis (Table 2). Notably, we concluded that the circRNF220 expression level at initial diagnosis is an independent and reliable predictor for relapse in pediatric patients with AML.

**Table 2:** Univariate and multivariate analyses for relapse in all pediatric AML patients (n=87)

Variable	Univariate Analysis		Multivariate Analysis	
	Hazard ratio(95%CI)	*P* Value	Hazard ratio(95%CI)	*P* Value
Age	1.034(0.876-1.225)	0.698	—	—
Gender(female *v* male)	0.39(0.103-1.472)	0.165	—	—
WBC	0.99(0.975-1.006)	0.212	—	—
Hemoglobin	1.035(1.007-1.064)	0.014	1.036(1.002-1.07)	0.037
Platelet	1(0.997-1.004)	0.950	—	—
BM blast (above *v* below median)	3.514(0.753-16.392)	0.110	—	—
Clinic risk(poor *v* inter-mediate *v* favorable)	3.037(0.931-9.904)	0.066	4.135(1.088-18.443)	0.043
One course to CR(no *v* yes)	1.958(0.995-3.854)	0.052	1.463(0.642-3.331)	0.365
circRNF220 expression (high *v* low)	5.749(1.219-27.101)	0.027	5.866(1.23-27.969)	0.026

NOTE. Hazard ratios greater than or less than 1 indicate an increased or decreased risk, respectively, of an event for the first category listed

Abbreviations: WBC white blood cells, FAB French-American-British, BM bone marrow, CR complete-remission

*P* value from univariate or multivariate Cox proportional hazards models

### CircRNF220 enhances the proliferation and impairs the apoptosis of human AML cells

Because circRNF220 was consistently upregulated in patients with primary and relapsed pediatric AML, we wondered whether circRNF220 alone is sufficient to induce leukemia progression. To evaluate the biological basis of circRNF220 dependency, we generated a vector for efficient expression of circRNF220 in AML cell lines (Supplementary Fig S3A-B). No effect on the RNF220 level was observed (Supplementary Fig S3C).

**Fig. 4:**
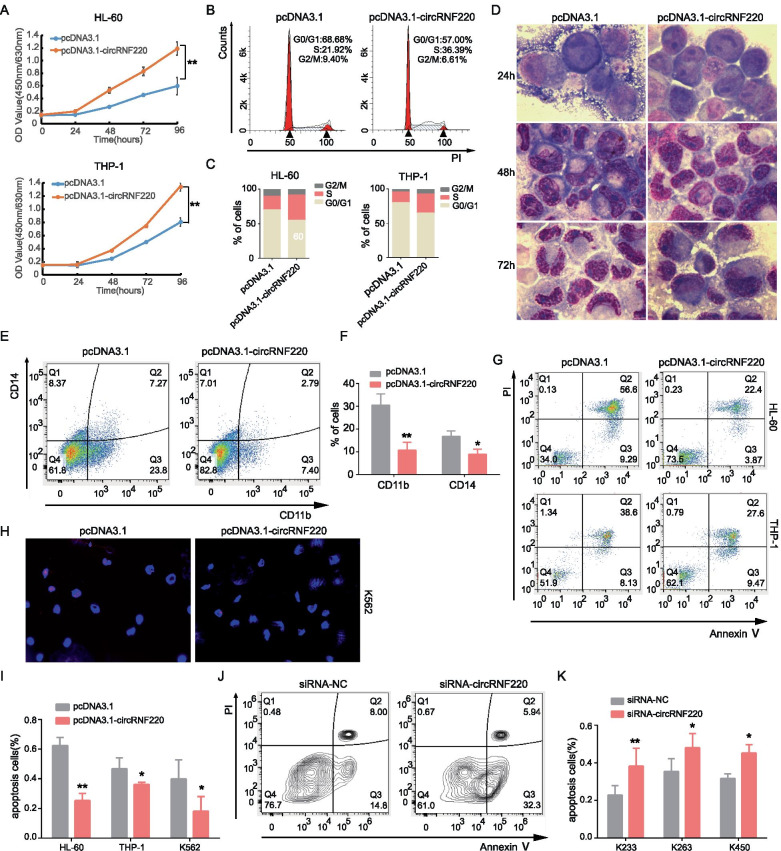
The requirement for circRNF220 in AML cells. **A** Effect of circRNF220 overexpression on the proliferation of HL-60 and THP-1 cells. **B**-**C** Flow cytometric analysis of cell cycle progression in AML cells. The numbers indicate the percentages of cells. **D** Microscopic analysis of Wright-Giemsa-stained cytospin preparations of THP-1 cells. Original magnification, 1000 × . **E** Flow cytometric analysis of myeloid differentiation of THP-1 cells. **F** The numbers indicate the percentages of differentiated cells. **G** Flow cytometric analysis of apoptosis in HL-60 and THP-1 cells. **H** Apoptosis was detected by TUNEL in K562 cells. **I** The numbers indicate the percentages of apoptotic cells in AML cell lines. **J** Flow cytometric analysis of apoptosis in primary AML patient cells. **K** The numbers indicate the percentages of apoptotic cells in primary AML BM cells. * *P* < 0.05, **P < 0.01

Next, we sought to investigate the effects of circRNF220 on leukemic cell proliferation and apoptosis. As shown in Fig. 4A, circRNF220 overexpression substantially accelerated the proliferation of AML cells. In addition, upregulation of circRNF220 significantly decreased the percentage of G0/G1-phase cells and increased the percentages of cells in S and G2/M phases (Fig. 4B-C). Notably, overexpression of circRNF220 induced a larger nucleo-cytoplasmic ratio and fewer lobulated nuclei which showed an immature like appearance (Fig. 4D), and downregulated CD11b and CD14 expression in THP-1 cells (Fig. 4E-F). A pronounced decrease in apoptosis was observed in AML cell lines by different assays (Fig. 4G-I). Interestingly, primary AML cells displayed a reduced total cell count and rapid apoptosis, as shown by staining for Annexin V, after circRNF220 KD (Fig. 4J-K, Supplementary Fig S3D-F). Together, these data showed the functional relevance of circRNF220 in the context of AML.

### Aberrant transcriptional networks regulated by circRNF220 in AML Cells

**Fig. 5:**
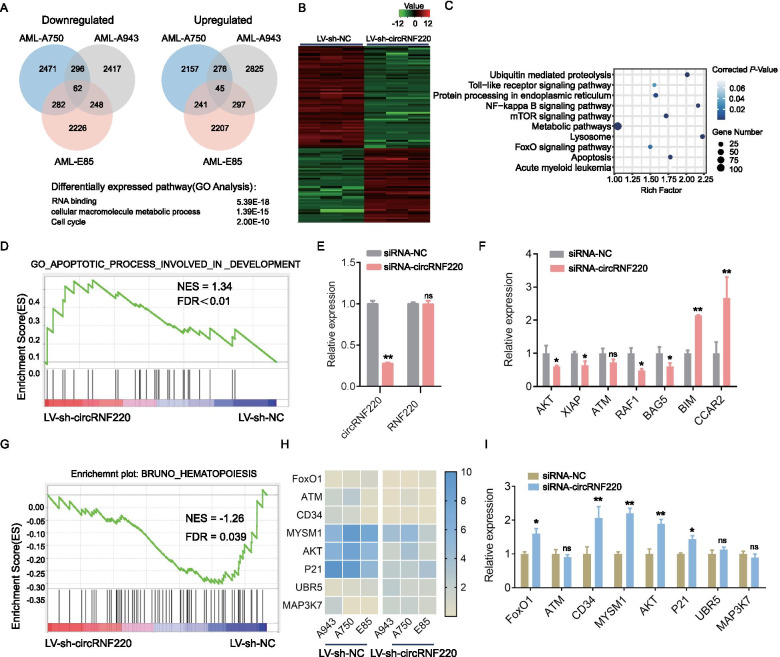
Transcriptional analysis of circRNF220 KD in AML patient cells. **A** Venn diagram indicating the numbers of significantly upregulated and downregulated genes in the AML-A750, AML-943, and AML-E85 patient samples based on RNA-seq 3 days post lentiviral infection with circRNF220 shRNA compared with scrambled control samples. **B** Heatmap of the top 100 differentially regulated genes in all three patients, representing data for replicates of cells expressing control (LV-sh-NA) or circRNF220 shRNA (LV-sh-circRNF220). **C** Kyoto Encyclopedia of Genes and Genomes (KEGG) pathway analysis of significantly dysregulated genes in the three samples. **D** Representative GSEA plot depicting apoptotic process involved in development. **E** Efficient circRNF220 KD in AML-K263 patient cells. **F** QRT-PCR analysis showing the expression of apoptosis-associated genes in cells from patient K263 with circRNF220 KD. The mean and SD values are expressed as percentages of GAPDH expression. **G** Representative gene set enrichment analysis (GSEA) plot depicting hematopoiesis. **H** Heatmap of differentially regulated relapse-related genes from RNA-seq data. **I** QRT-PCR analysis showing the expression of relapse associated genes in HL-60 cells with circRNF220 overexpression. The mean and SD values are expressed as percentages of GAPDH expression. NES, normalized enrichment score, FDR, false discovery rate. *P < 0.05, **P < 0.01, ns: not significant

To define the transcriptional functions of circRNF220 in AML cells, we performed gene expression analyses by RNA-seq in three pediatric AML patients’ primary cells after LV-sh-circRNF220 infection (Supplementary Fig S3G-H). A total of 107 mRNAs (62 downregulated and 45 upregulated) were significantly modulated in all three samples (fold change > 1.5 or < 0.67 and false discovery rate < 0.05) by circRNF220 KD (Fig. 5A-B).

To identify the molecular pathways regulated by circRNF220, we performed Kyoto Encyclopedia of Genes and Genomes (KEGG) analysis on the commonly dysregulated transcripts. We observed that genes involved in apoptosis, lysosome and acute myeloid leukemia were positively enriched (Fig. 5C), consistent with their functions. We also applied GSEA to identify pathways differentially regulated by loss of circRNF220 and found statistically significant dysregulation of several GSEA pathways common to all three AML patients. The term apoptotic process involved in development also had a significant *P* value and a normalized enrichment score of 1.34 (Fig. 5D). We independently evaluated several apoptosis-related genes in AML-E697 cells (Fig. 5E) and found that the expression of genes, which inhibit apoptosis, including AKT, XIAP, ARF1 and BAG5 were significantly downregulated, pro-apoptotic gene BIM was upregulated (Fig. 5F). Strikingly, we also showed that hematopoiesis had a normalized enrichment score of -1.26 (Fig. 5G). Then we wondered whether circRNF220 is responsible for the enrichment or maintenance of clonal population in AML relapse, a potential gene profile associated with recurrence of AML were investigate through conducting systematic reviews of the literature [28–33]. In agreement with the KEGG analysis results of molecular pathways regulated by circRNF220, lots of relapse-related genes were significantly enriched in NF-κB signaling pathway, mTOR signaling pathway, and FoxO signaling pathway (Fig. 5C). We verified the differentially expressed genes implicated in relapse from our transcriptome data in HL-60 overexpression system (Fig. 5H-I).A number of genes, such as CD34, FoxO1, MYSM1, AKT1 and P21, which have potential roles in hematopoietic stem cell function and chemotherapy resistant were uncovered [34–38]. Collectively, these data indicated that circRNF220 played an essential role in cell apoptosis and AML relapse.

### CircRNF220 acts as a sponge for miR-30a

**Fig. 6:**
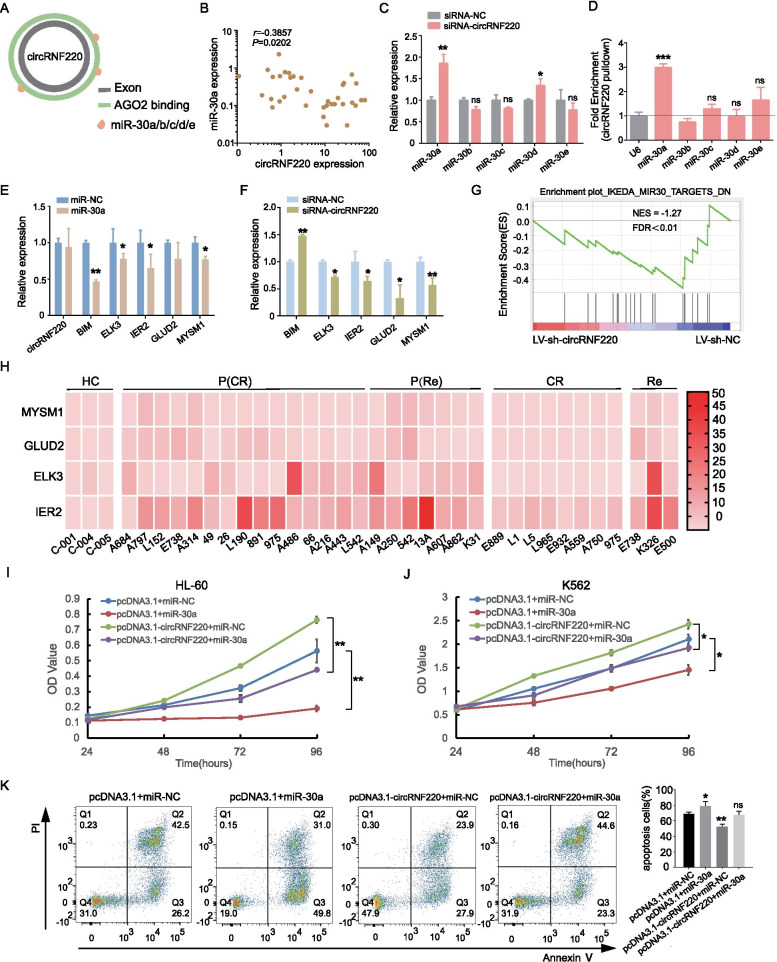
MiR-30a partially rescues circRNF220 function. **A** Illustration of miRNA-binding sites in circRNF220; miRNAs predicted to interact with circRNF220 are highlighted in orange. **B** Correlation between circRNF220 and miR-30a levels. **C** The miR-30 family expression levels in siRNA-circRNF220 were determined by qPCR in primary AML cells. **D** RNA pulldown using biotinylated circRNF220 and detection of miRNAs in primary AML cells. U6 was used as the control. Plots with error bars show the mean ± SD from triplicates unless otherwise stated. **E** Abundances of circRNF220, BIM, ELK3, IER2, GLUD2, and MYSM1 upon overexpression of miR-30a in primary AML cells. GAPDH was used as the control. **F** Abundances of BIM, ELK3, IER2, GLUD2, and MYSM1 upon circRNF220 knockdown in primary AML cells. **G** Representative GSEA plots showing the most strongly downregulated miR-30 targets (NES = -1.27, FDR < 0.001) in primary AML patient cells treated with LV-sh-circRNF220. The results were compared with those in AML patient cells treated with LV-siRNA-NC. **H** Relative transcript levels of miR-30 target mRNAs in AML patient cells and normal control cells, normalized to GAPDH transcript levels. The heatmap indicates the average of three independent experiments. **I**-**J** Cell proliferation decreased upon miR-30a OE and was rescued after circRNF220 overexpression in HL-60 (**I**) and K562 cells (**J**). The data are presented as the mean values of triplicates. **K** Cell apoptosis upon miR-30a mimics and rescued after circRNF220 overexpression in HL-60 cells. The numbers indicate the percentages of apoptotic HL-60 cells. Data represent mean value from triplicates. HC, healthy control; P (CR), primary patient with complete remission; P (Re), primary patient with relapse; CR, complete remission; Re, relapse; * *P* < 0.05, **P < 0.01, *** *P* < 0.001. ns: not significant

As circRNAs often function by interacting with miRNAs, we searched for candidate miRNAs interacting with circRNF220. Through computational prediction, we identified five candidates (miR-30a/b/c/d/e). Their predicted binding sites were located within highly conserved AGO-binding sites in circRNF220 (Fig. 6A). Using miRNA expression data from a subset (n = 36) of our cohort, we analyzed the correlations between the expression of these miRNAs and that of circRNF220. The expression of only one miRNA, miR-30a, was significantly correlated with circRNF220 expression (Fig. 6B; Supplementary Figure S4A-D). As expected, circRNF220 KD increased the miR-30a abundance in AML cells (Fig. 6C), while circRNF220 OE decreased its abundance (Supplementary Fig S4E). Subsequently, we performed an RNA pulldown assay and observed significant enrichment of miR-30a in primary AML cells and AML cell lines (Fig. 6D; Supplementary Fig S4F). We then enhanced the expression of miR-30a with mimics (Supplementary Fig S4G) and found that miR-30a OE in AML cells significantly decreased the abundances of EKL3 and IER2, well-characterized oncogenes, compared with those in negative control cells (Fig. 6E). Consistent with the upregulation of miR-30a upon circRNF220 KD, the abundances of ELK3 and IER2 were significantly decreased (Fig. 6F). Similarly, other target genes, such as, GLUD2, and MYSM1, were downregulated in both miR-30a OE cells and circRNF220 KD cells, except BIM (Fig. 6E-F).

We next applied GSEA to identify pathways differentially regulated by circRNF220 inhibition and found significant downregulation of miR-30 targets, identical to the above findings (Fig. 6G). To understand the relationship between circRNF220 and miR-30a targets in AML samples, we determined the relative abundances of those genes with respect to GAPDH in our panel of samples from 22 primary AML patients, 8 AML patients with complete remission, 3 relapse patient and 3 healthy controls. ELK3, IER2, GLUD2 and MYSM1 showed consistently higher relative abundances in the evaluated samples from primary AML patients and relapse patients than that in the samples from patients with CR or the healthy controls (Fig. 6H). Particularly, there is a significantly negative correlation between miR-30a expression and MYSM1, GLUD2 and IER2 (Supplementary Fig S4E), and a significantly positive correlation between circRNF220 expression and MYSM1 and IER2 were observed subsequently (Supplementary Fig S4F). Furthermore, OE of miR-30a significantly attenuated the proliferation which induced by enhancing circRNF220 expression in HL-60 and K562 cells (Fig. 6I-J). Predictively, cell proliferation increased upon inhibitor-miR-30a and was impaired after circRNF220 KD in HL-60 cells (Supplementary Fig S4G). Importantly, HL-60 cells treated with miR-30a showed evidence of apoptosis, and the significant increase in the apoptosis of HL-60 cells induced by OE of circRNF220 was restored by transfection with miR-30a (Fig. 6K). In addition, inhibiting miR-30a arrested primary pediatric AML cell and HL-60 cells apoptosis, as measured by Annexin V staining, which effects were eliminated by treatment with siRNA-circRNF220 (Supplementary Fig S4H-J). Collectively, these data suggest that circRNF220 may function as an endogenous miR-30a sponge to sequester miR-30a and inhibit its activity, which results in increased levels of its downstream targets, including MYSM1 and IER2.

## Discussion

Tumor-specific gene expression patterns have been widely researched for their potential roles in cancer diagnosis and prognosis [39–43]. Relapses occur in exceeding30% of children with AML, and the molecular mechanisms underlying AML relapse are still not fully understood [44, 45]. In the current study, we demonstrated that the differential expression patterns of circRNF220 in PB or BM were able to distinguish pediatric patients with AML from individuals with other hematological diseases with extremely high specificity and sensitivity. Furthermore, the circRNF220 expression level at diagnosis could predict relapse in pediatric AML patients. Importantly, depletion of circRNF220 significantly suppressed cell cycle progression and increased apoptosis in AML cell lines and primary pediatric AML BM cells. The combined results of transcriptome analysis and molecular experiments substantiated that circRNF220 activated apoptotic signaling pathways by regulating miR-30a and its targets. Our inspiring findings highlighted the importance of circRNF220 in regulating the activities of leukemic cells, suggesting that accumulation of circRNF220 might be a potential biomarker for diagnosis and predicting recurrence in pediatric AML.

CircRNAs constitute a novel class of exceptionally stable RNA species in eukaryotes. Some are abundant and evolutionarily conserved and are anticipated to be used as biomarkers in diseases [46, 47]. For example, circ-ANXA2 is upregulated in AML cell lines and may be a potential biomarker and therapeutic target in AML [20]. Lei et al*.* found that circ_0009910 is significantly upregulated in AML BM samples and might be a novel prognostic biomarker in AML [48]. A new study reported that hsa_circ_0012152 can discriminate ALL from AML in adult patients, consistent with our results in the pediatric population [49]. Moreover, we found that circRNF220 not only showed excellent performance in distinguishing AML from ALL but also allowed clear separation of AML from other hematological diseases, including MDS and CML. Of note, the level of circRNF220 in PB samples also had outstanding specificity and sensitivity for the differential diagnosis of pediatric AML. More importantly, the research indicated that the relative circRNF220 expression level at diagnosis could be an independent prognostic indicator for childhood AML relapse.

Strikingly, these findings have crucial implications for improving the diagnostic yield of biopsies from patients whose BM sample yield or quality is inadequate for accurate histological diagnosis [50–52]. BM aspiration and biopsy are often relatively time-consuming and unpleasant experiences, especially for pediatric patients. In contrast, only a small amount of PB is needed to obtain an adequate amount of RNA for detecting circRNF220, possibly because of its high stability and remarkable abundance. However, the sample size was still limited, the results need to be interpreted with caution and more validation.

We noted that circRNF220 can radically promote proliferation, accelerate cell cycle progression and suppress apoptosis in AML cell lines and primary pediatric AML BM cells. Gene expression analysis of primary AML BM cells treated with LV-siRNA-circRNF220 indicated a complex phenotype including genes related to the terms hematopoiesis and apoptotic process involved in development. These phenotypes were in accordance with those observed in in vitro experiments.

CircRNAs are known to act as miRNA sponges [53–56]. Guo et al. predicted that hsa_circ_0012152 might be involved in AML through the miR-491-5p/EGFR/MAPK1 axis or the miR-512-3p/EGFR/MAPK1 axis [49]. Because internal ribosome entry sites were not found in the region upstream of a putative open reading frame in circRNF220, we first considered that this circRNA functions as a competing endogenous RNA. By combining transcriptomic and experimental data, we provide evidence that miR-30a is a key downstream effector of circRNF220 in our models. Although miR-30a is a crucial regulator of human cancer progression, few studies on the function and mechanism of AML have been conducted to date [57]. Our results revealed that miR-30a can partially offset the functions of circRNF220 through targets MYSM1 and IER2. Many lines of evidence support the essential role of MYSM1 in hematopoiesis and hematopoietic stem cells [58], and suggested that it might be related to the recurrence of AML. But little is known about the function of IER2 in AML, Neeb et al. showed IER2 is strongly upregulated in a wide variety of human tumors, and is a new player in the regulation of tumor progression and metastasis [59]. Either way, these data indicated that circRNF220 regulating cell growth and associated with relapse partly relied on the miR-30a/MYSM1 and miR-30a/IER2 pathway.

Concurrent to miRNA sponge, regulation of RBPs is a plausible alternative role for circRNAs, and interactions of circRNAs with RBPs have been reported [18, 60]. This may be a possible reason behind only a partial rescue by miR-30a and not complete remission of circRNF220 effects in Fig. 6I-K and Supplementary Fig S4G-J. Thus, we utilized a web tool, CircInteractome, to explore potential interactions of circRNF220 with RBPs [26, 61, 62]. Except Ago2 protein, EIF4A3, PTB, and TDP43 are predicted to bind circRNF220 based on sequence matches. The impact of potential secondary or tertiary structures on circRNA sequence available for interaction with RBPs cannot be considered systematically, hence experimental validation is essential to verify the predicted RBPs in the future.

Given that circRNAs are resistant to RNA exonucleases, they have a sufficiently long half-life within cells [12]. Although 4 circRNAs can be generated from the *RNF220* mRNA by variable cyclization, only circRNF220 was highly expressed in childhood AML. RNF220 is a RING domain E3 ubiquitin ligase that was reported to mediate the ubiquitination of multiple targets and is involved in various developmental and disease progression [63–65]. It is intriguing that circRNF220 may also play a vital role in the regulation of cellular protein metabolic processes and polyubiquitination (GO analysis and GSEA results), although circRNF220 had a unique expression profile distinct from that of the *RNF220* gene. We observed only a weak correlation between circRNF220 and RNF220, and it is unclear whether competition between canonical splicing and back splicing is likely to exist for the majority of loci that generate circRNF220.

In summary, circRNF220 was differentially expressed within distinct subtypes that compose the pediatric AML hierarchy, with definite prognostic value in relapse. The circRNF220-miR-30a axis operates in the progression of AML by facilitating the expression of its targets related to apoptosis. Treatment with siRNA targeting circRNF220 suppressed the growth and survival of AML cells. Collectively, these data suggest that circRNF220 should be investigated as a potentially effective biomarker and therapeutic target in pediatric AML.

## Supplementary Information

**Additional file 1: Supplementary Fig S1.** Validation the expression levels of circRNA in pediatric AML. (A) QRT-PCR results of circRNA array validation. (B) The expression levels of circRNAs identified in RNF220 gene locus. (C) The expression level of RNF220 in each patient was detected. (D) A weak positive correlation between the levels of circRNF220 and RNF220 were observed in pediatric AML specimens (*r*=0.2395, *P*=0.0255). (E) Comparing the expression of circRNF220 and its linear cognate gene RNF220 in pediatric AML, normalized to the expression of GAPDH and presented as the 2-△^Ct^ value. *P < 0.05, **P < 0.01, ***P < 0.001. ns: not significant.

**Additional file 2: Supplementary Fig S2.** ROC analysis for circRNF220 in bone marrow of AML patients. (A) The AUC indistinguishing AML and normal individual was 0.9735. (B) The AUC indistinguishing AML and ALL was 0.9209. (C) The AUC indistinguishing AML and other hematologic malignancies was 0.9274. (D)Spearman’s coefficient scatters plot of the fold-changes of circRNF220 in the BM and PB samples (*r*=0.8107, *P*<0.0001). BM: bone marrow, PB: peripheral blood.

**Additional file 3: Supplementary Fig S3.** Relative level of circRNF220 in AML cells lentiviral infected with circRNF220 shRNA or circRNF220 overexpression vector. (A) Schematic of the circRNF220 overexpression vector. (B) Efficient overexpression of circRNF220 in HL-60, THP-1 and K562 cell lines. (C) circRNF220 overexpression vector did not influence parental gene expression in HL-60, THP-1 and K562 cell lines. (D) Schematic representation of the sites of the siRNA specific to the back-splice junction of circRNF220. (E) Efficient knockdown of circRNF220 in AML-K233, AML-K262, and AML-K450 three patients’ cells. (F) Specific siRNA-circRNF220 did not influence parental gene expression in AML-K233, AML-K262, and AML-K450 three patients’ cells. (G) Efficient knockdown of circRNF220 in AML-A750, AML-943, and AML-E85 three patients’ cells. (H) Specific siRNA-circRNF220 did not influence parental gene expression in AML-A750, AML-943, and AML-E85 three patients’ cells. *P < 0.05, **P < 0.01, ***P < 0.001. ns: not significant.

**Additional file 4: Supplementary Fig S4.** CircRNF220 regulates AML proliferation and apoptosis through targeting miR-30a. (A) Correlation between circRNF220 and miR-30b, miR-30c, miR-30d, miR-30e, respectively. (B) The miR-30 family expression levels in overexpression circRNF220 by real-time PCR in HL-60. (C) RNA pulldown using biotinylated circRNF220 and detection of miR-30a in HL-60. (D) miR-30 family expression levels in AML cells transfected with miR-30a. (E) Correlation between miR-30a and MYSM1, GLUD2, ELK3, and IER2, respectively. (F) Correlation between circRNF220 and MYSM1, GLUD2, ELK3, and IER2, respectively. (G) Cell proliferation increased upon inhibitor-miR-30a and was impaired after circRNF220 KD in HL-60 cells. (H) Apoptosis was arrested by miR-30a inhibitor and which effect was eliminated after circRNF220 KD in HL-60 cells. The numbers indicate the percentages of apoptotic cells. The data are presented as the mean values of triplicates. (I) The relative expression of circRNF220 and miR-30a in AML primary cells when transfected different small interfering RNA. (J) Apoptosis was arrested upon miR-30a inhibitor treatment, and this effect was eliminated after circRNF220 siRNA transfection in primary pediatric AML cells. The numbers indicate the percentages of apoptotic primary AML cells. The data are presented as the mean values of triplicates. *P < 0.05, **P < 0.01, ***P < 0.001. ns: not significant.

**Additional file 5: Supplementary Table S1.** Detailed characteristics of pediatric patients with AML (*n*=149).

**Additional file 6: Supplementary Table S2.** Detailed characteristics of pediatric patients with newly diagnosed Hematologic Disorders except for AML (*n*=147).

**Additional file 7: Supplementary Table S3.** Sequences of the PCR primers in this study (5’-3’).

## Data Availability

The datasets used and/or analyzed during the current study are available from the corresponding author on reasonable request.

## Abbreviations

CircRNA: Circular RNA
AML: Acute myeloid leukemia
ALL: Acute lymphoblastic leukemia
WBC: White cell count
MRD: Minimal residual disease
MYSM1: Myb like, SWIRM and MPN domains 1
IER2: Immediate early response 2
KD: Knockdown
OE: Overexpression
BM: Bone marrow
PB: Peripheral blood
FAB: French-American-British
ITP: Idiopathic thrombocytopenic purpura
MDS: Myelodysplastic syndrome
CML: Chronic myeloid leukemia
JMML: Juvenile type monocyte leukemia
qRT-PCR: Real-time quantitative polymerase chain reaction
FISH: Fluorescence in situ hybridization
FBS: Fetal bovine serum
siRNA: Small interfering RNAs
TUNEL: Terminal Deoxynucleotidyl Transferase (TdT)-Mediated dUTP Nick-End Labeling
RNA-seq: RNA sequencing
GSEA: Gene Set Enrichment Analysis
ROC curve: Receiver operating characteristic curve
AUC: Area under curve
RFS: Relapse-free survival
CR: Complete remission
KEGG: Kyoto Encyclopedia of Genes and Genomes
