# Supplementary figures and images for: CircRNF220, not its linear cognate gene RNF220, regulates cell growth and is associated with relapse in pediatric acute myeloid leukemia
Source: Mol Cancer. 2021 Oct 26;20:139. doi: 10.1186/s12943-021-01395-7 (PMC8549339; doi:10.1186/s12943-021-01395-7)

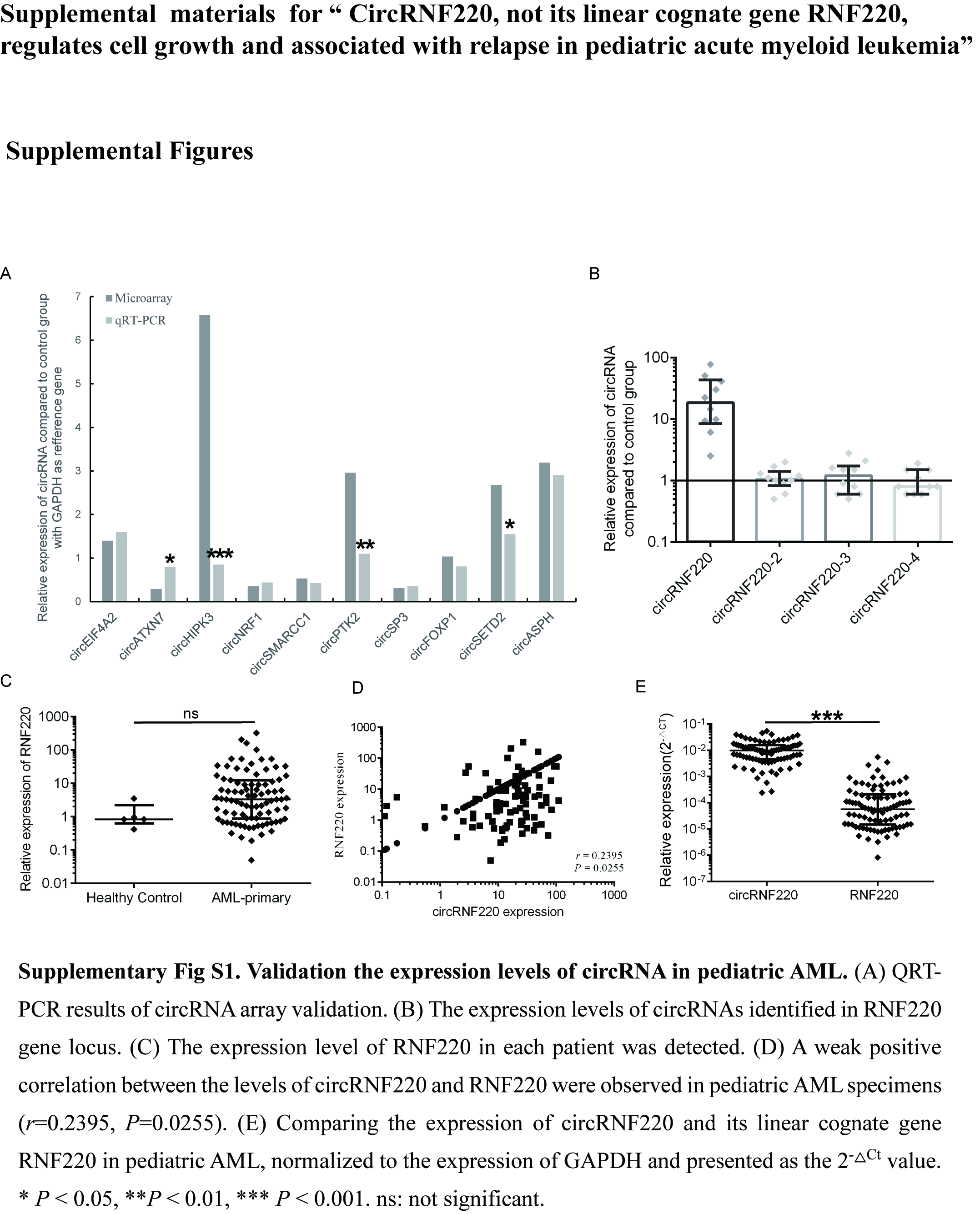

Supplement: Supplementary file 1 — Additional file 1: Supplementary Fig S1. Validation the expression levels of circRNA in pediatric AML. (A) QRT-PCR results of circRNA array validation. (B) The expression levels of circRNAs identified in RNF220 gene locus. (C) The expression level of RNF220 in each patient was detected. (D) A weak positive correlation between the levels of circRNF220 and RNF220 were observed in pediatric AML specimens (r=0.2395, P=0.0255). (E) Comparing the expression of circRNF220 and its linear cognate gene RNF220 in pediatric AML, normalized to the expression of GAPDH and presented as the 2-△Ct value. *P < 0.05, **P < 0.01, ***P < 0.001. ns: not significant. [file 12943_2021_1395_MOESM1_ESM.tif]

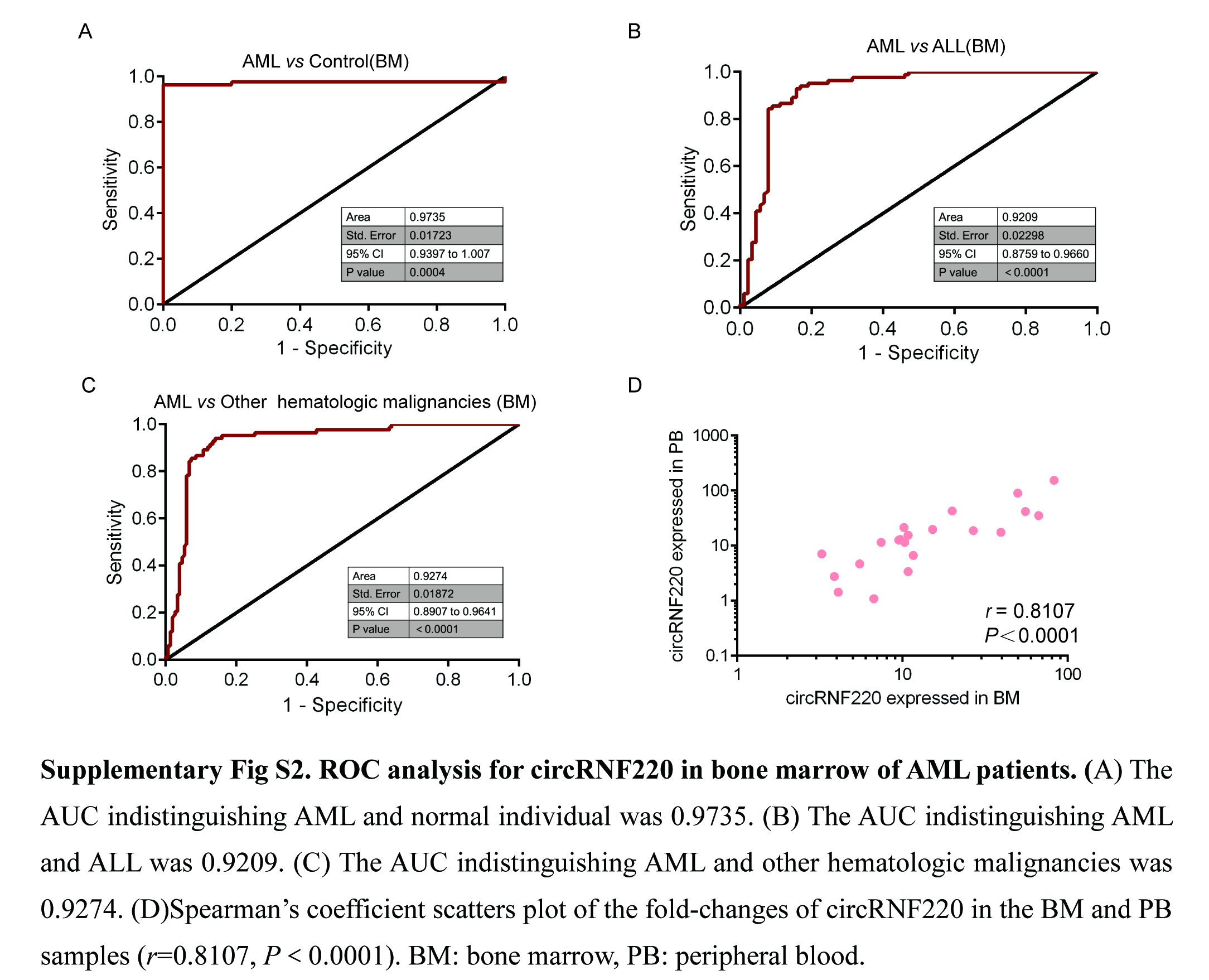

Supplement: Supplementary file 2 — Additional file 2: Supplementary Fig S2. ROC analysis for circRNF220 in bone marrow of AML patients. (A) The AUC indistinguishing AML and normal individual was 0.9735. (B) The AUC indistinguishing AML and ALL was 0.9209. (C) The AUC indistinguishing AML and other hematologic malignancies was 0.9274. (D)Spearman’s coefficient scatters plot of the fold-changes of circRNF220 in the BM and PB samples (r=0.8107, P<0.0001). BM: bone marrow, PB: peripheral blood. [file 12943_2021_1395_MOESM2_ESM.tif]

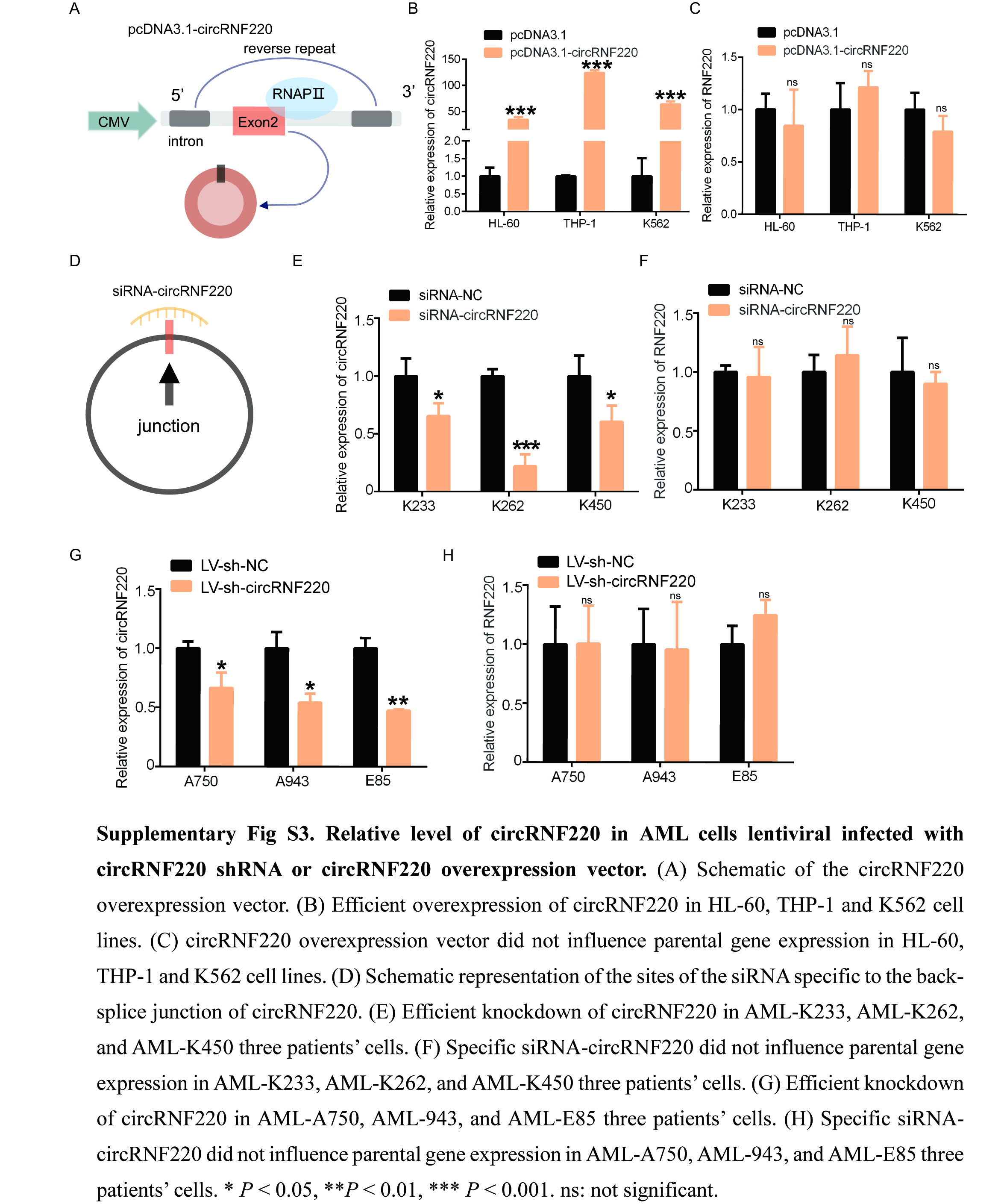

Supplement: Supplementary file 3 — Additional file 3: Supplementary Fig S3. Relative level of circRNF220 in AML cells lentiviral infected with circRNF220 shRNA or circRNF220 overexpression vector. (A) Schematic of the circRNF220 overexpression vector. (B) Efficient overexpression of circRNF220 in HL-60, THP-1 and K562 cell lines. (C) circRNF220 overexpression vector did not influence parental gene expression in HL-60, THP-1 and K562 cell lines. (D) Schematic representation of the sites of the siRNA specific to the back-splice junction of circRNF220. (E) Efficient knockdown of circRNF220 in AML-K233, AML-K262, and AML-K450 three patients’ cells. (F) Specific siRNA-circRNF220 did not influence parental gene expression in AML-K233, AML-K262, and AML-K450 three patients’ cells. (G) Efficient knockdown of circRNF220 in AML-A750, AML-943, and AML-E85 three patients’ cells. (H) Specific siRNA-circRNF220 did not influence parental gene expression in AML-A750, AML-943, and AML-E85 three patients’ cells. *P < 0.05, **P < 0.01, ***P < 0.001. ns: not significant. [file 12943_2021_1395_MOESM3_ESM.tif]

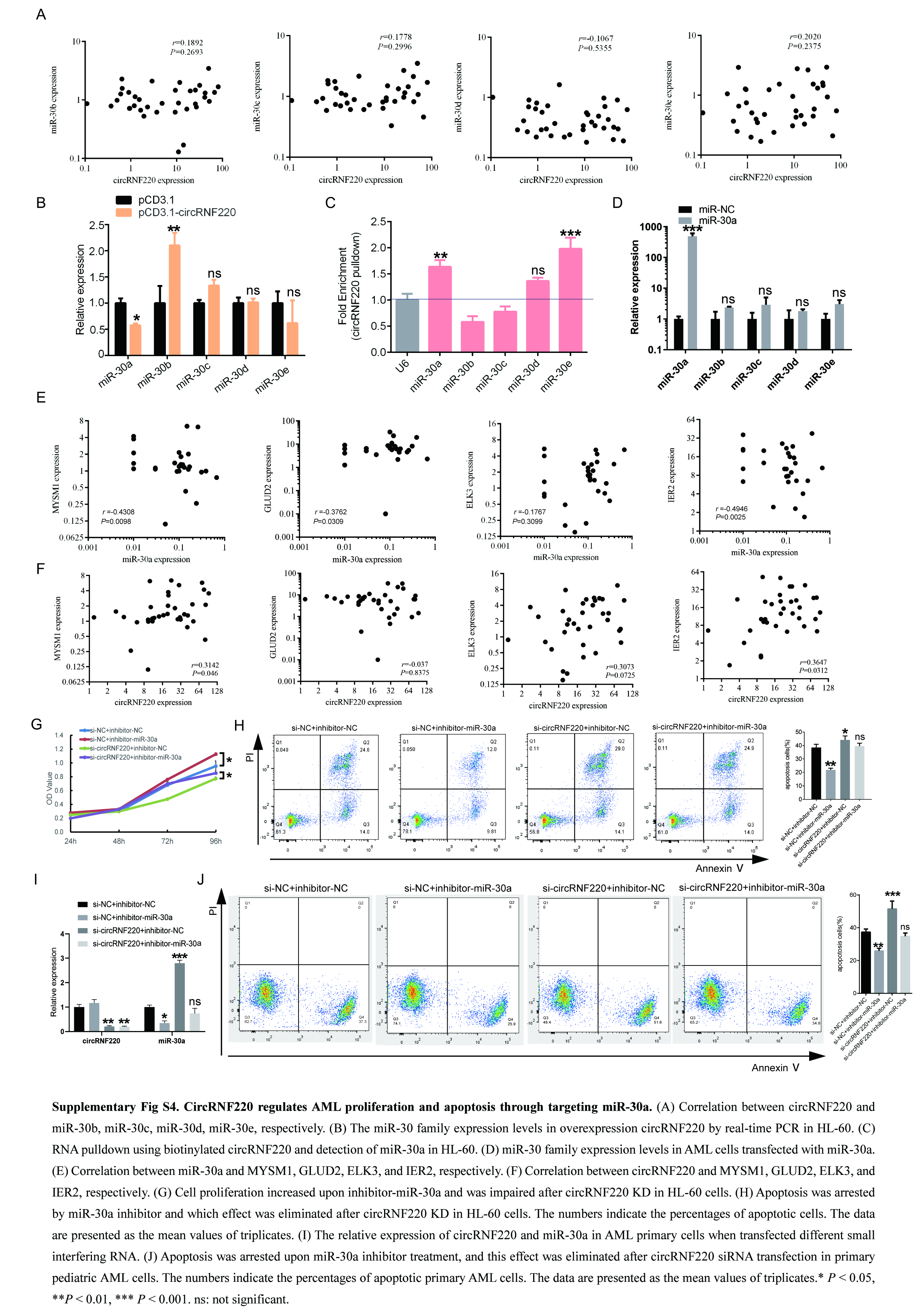

Supplement: Supplementary file 4 — Additional file 4: Supplementary Fig S4. CircRNF220 regulates AML proliferation and apoptosis through targeting miR-30a. (A) Correlation between circRNF220 and miR-30b, miR-30c, miR-30d, miR-30e, respectively. (B) The miR-30 family expression levels in overexpression circRNF220 by real-time PCR in HL-60. (C) RNA pulldown using biotinylated circRNF220 and detection of miR-30a in HL-60. (D) miR-30 family expression levels in AML cells transfected with miR-30a. (E) Correlation between miR-30a and MYSM1, GLUD2, ELK3, and IER2, respectively. (F) Correlation between circRNF220 and MYSM1, GLUD2, ELK3, and IER2, respectively. (G) Cell proliferation increased upon inhibitor-miR-30a and was impaired after circRNF220 KD in HL-60 cells. (H) Apoptosis was arrested by miR-30a inhibitor and which effect was eliminated after circRNF220 KD in HL-60 cells. The numbers indicate the percentages of apoptotic cells. The data are presented as the mean values of triplicates. (I) The relative expression of circRNF220 and miR-30a in AML primary cells when transfected different small interfering RNA. (J) Apoptosis was arrested upon miR-30a inhibitor treatment, and this effect was eliminated after circRNF220 siRNA transfection in primary pediatric AML cells. The numbers indicate the percentages of apoptotic primary AML cells. The data are presented as the mean values of triplicates. *P < 0.05, **P < 0.01, ***P < 0.001. ns: not significant. [file 12943_2021_1395_MOESM4_ESM.tif]
