# Supplementary material for: CircRNF220, not its linear cognate gene RNF220, regulates cell growth and is associated with relapse in pediatric acute myeloid leukemia
Source: Mol Cancer. 2021 Oct 26;20:139. doi: 10.1186/s12943-021-01395-7 (PMC8549339; doi:10.1186/s12943-021-01395-7)
Supplement: Supplementary file 7 — Additional file 7: Supplementary Table S3. Sequences of the PCR primers in this study (5’-3’). [file 12943_2021_1395_MOESM7_ESM.doc]

**Supplementary Table S3** **Sequences of the PCR primers in this study (5’-3’)**

| **Oligonucleotide sets used for mimics，small interfering RNAs and circRNA** | |
| --- | --- |
| miR-NC | GenePharma Biotech |
| miR-30a | GenePharma Biotech |
| Control Probe | Exonbio Lab |
| circRNF220 Probe | 5’-CATTCCCTCCTAAGACATCTGTCATTC-3’ |
| siRNA-NC | RiboBio |
| siRNA-circRNF220 | 5’-AUGACAGAUGUCUUAGGAGdTdT-3’ (sense)  5'-CUCCUAAGACAUCUGUCAUdTdT-3' (antisense) |
| **Primers for real-time PCR of microRNAs, circRNAs and mRNA** | |
| hsa-miR-30a-5p-F | CAGTGCTGTGTAAACATCCTCG |
| hsa-miR-30a-5p-R | TATGGTTGTTCACGACTCCTTCAC |
| hsa-miR-30b-F | GCTGCCGTTGTAAACATCCTAC |
| hsa-miR-30b-R | CAGAGCAGGGTCCGAGGTA |
| hsa-miR-30c-F | TGCTGCCGTGTAAACATCCTA |
| hsa-miR-30c-R | AGAGCAGGGTCCGAGGAT |
| hsa-miR-30d-F | TCCGTGTAAACATCCCCG |
| hsa-miR-30d-R | CAGAGCAGGGTCCGAGGTA |
| hsa-miR-30e-F | GCTTGCCGTGTAAACATCCTT |
| hsa-miR-30e-R | CACTTCCTCTGCACTTGTTGGTAT |
| U6-F | CGCTTCGGCAGCACATATAC |
| U6-R | TTCACGAATTTGCGTGTCAT |
| circRNF220-F(Divergent) | TGCTGTCTCTGGCCTCATTTC |
| circRNF220-R(Divergent) | GGGAATCATTCCCTCCTAAGAC |
| circRNF220-F(convergent) | CCTTTCACCAACGGTTCCTA |
| circRNF220-R(convergent) | ATCTTCTTCCCAGTGTCATCAA |
| circRNF220-F(mouse) | GATGCAGATGGCAAGGAATA |
| circRNF220-R(mouse) | TGGTGAAGGGAATGTGAACG |
| circEIF4A2-F | GACACTGACCATTACACAGGCT |
| circEIF4A2-R | AGTGTATCCAACTTCCATTCCA |
| circATXN7-F | CTCTGCCCAGTCCTGAAGTG |
| circATXN7-R | ATTCTTTCCGCTCCTTCCCG |
| circHIPK3-F | GCTTTCAGCACCGTAACCATACT |
| circHIPK3-R | GGAATACACAACTGCTTGGCTCT |
| circNRF1-F | GTCCTCTGTATCTCACCCTCCA |
| circNRF1-R | CGTGTTCCTCCATGAAGTTCTACT |
| circSMARCC1-F | AGCGGCTTTGGAGGAGTT |
| circSMARCC1-R | TTTCGATATGCCAAGTATCAAG |
| circPTK2-F | GGAAAAAGAGGAAAGATTTCTGC |
| circPTK2-R | AAGAGATGCCTGACCTGGATAG |
| circSP3-F | AGAGTCTCAGCAGCCAACCA |
| circSP3-R | GGCAACTGACCAGTGCTTAGA |
| circFOXP1-F | ATGAACCCACATGCCTCTAC |
| circFOXP1-R | CCACCTGCTGTTGCTCTTTA |
| circSETD2-F | AGGACAGACATGTCCAACAGT |
| circSETD2-R | GGGGCAGGTGTTTGATCTCT |
| circASPH-F | TGATGCTGATGGTGATGGAGAT |
| circASPH-R | AGTTCCTGAGAGTCCGCCTT |
| circRNF220-2-F | GGCATCACCCCACTCATCTG |
| circRNF220-2-R | CATATGCTCCTGCAGCTCACT |
| circRNF220-3-F | GAGAGTCTCCAACGGCATCAC |
| circRNF220-3-R | ACAACAGGAGGGACTTTGGG |
| circRNF220-4-F | AGGAGAGAGAGGCACTTCGG |
| circRNF220-4-R | TCTGCCCTTCATCTTGCTTCC |
| GAPDH-F | GCTGAACGGGAAGCTCACTG |
| GAPDH-R | GTGCTCAGTGTAGCCCAGGA |
| GAPDH-F(convergent) | AGAAGGCTGGGGCTCATTTG |
| GAPDH-R(convergent) | AGGGGCCATCCACAGTCTTC |
| GAPDH-F(Divergent) | GAAGACTGTGGATGGCCCCT |
| GAPDH-R(Divergent) | CAAATGAGCCCCAGCCTTCT |
| RNF220-F | TCTCTCACGCCAACTGAGTC |
| RNF220-R | CTCCTAAGACTTGCCGTGGA |
| AKT-F | GGGGTTTCTCCCAGGAGGTT |
| AKT-R | GTCCATGGTGTTCCTACCCA |
| XIAP-F | ACGAAGGGACTTCCGTTTCC |
| XIAP-R | GGACTTGTCCACCTTTTCGC |
| ATM-F | TTACGGGTGTTGAAGGTGTCT |
| ATM-R | GGATTCATGGTCCAGTCAAAG |
| RAF1-F | GAACAGTGGTCAATGTGCGA |
| RAF1-R | TTCAGGAACGTCTTCCGAGC |
| BAG5-F | CAGACTTCTTTTGGTGCTTGTG |
| BAG5-R | TCTGACAGACCACTGAAGCC |
| BCL2L11-F | GTATTCGGTTCGCTGCGTTC |
| BCL2L11-R | CGCAGGCTGCAATTGTCTAC |
| CCAR2-F | AACGAAGGAATGTCATCGGGG |
| CCAR2-R | AAGCTGTGCCTGAGAAGTTG |
| ELK3-F | TGTCAGCATGGAAAGTCGGG |
| ELK3-R | GCACTCTCCATACCCAGATGT |
| IER2-F | GTGTCGGAGTTCTGTCTGGG |
| IER2-R | CCAAACACTCATTGCCCGTG |
| GLUD2-F | ACCTTGACTCAGTGAATGCCT |
| GLUD2-R | TGCTGGACAACAATACGTGA |
| MYSM1-F | CCCAGATGGCTCTTATCGCTT |
| MYSM1-R | TTTATCCATGGGGACGCTGC |
| CD34-F | GGAGAAAGGCTGGGCGAAG |
| CD34-R | CACCACGTGTTGTCTTGCTG |
| FOXO1-F | AGGGTTAGTGAGCAGGTTACAC |
| FOXO1-R | CTGCACACATTGGGCAAACA |
| UBR5-F | GCACATGGACGGGAAAATGG |
| UBR5-R | CGCTTTCGGTTTTCCTGCTG |
| P21-F | GCAGACCAGCATGACAGATTT |
| P21-R | GATGTAGAGCGGGCCTTTGA |
| MAP3K7-F | TGGTGCTGAACCATTGCCA |
| MAP3K7-R | TGCAACCAGCAGTAAGTTTGG |

F: Forward Prime；R, Reverse Prime.
